# Therapeutics Development for Pseudoxanthoma Elasticum and Related Ectopic Mineralization Disorders: Update 2020

**DOI:** 10.3390/jcm10010114

**Published:** 2020-12-31

**Authors:** Hongbin Luo, Qiaoli Li, Yi Cao, Jouni Uitto

**Affiliations:** 1 Department of Dermatology and Cutaneous Biology, Sidney Kimmel Medical College and the PXE International Center for Excellence in Research and Clinical Care, Thomas Jefferson University, Philadelphia, PA 19107, USA; luohongbin43@126.com (H.L.); Qiaoli.Li@Jefferson.edu (Q.L.); 2 Department of Dermatology, The First Affiliated Hospital of Zhejiang Chinese Medical University, Hangzhou 310006, China; caoyi1965@163.com; 3 Jefferson Institute of Molecular Medicine, Thomas Jefferson University, Philadelphia, PA 19107, USA

**Keywords:** ectopic mineralization disorders, pseudoxanthoma elasticum, generalized arterial calcification of infancy, arterial calcification due to CD73 deficiency, therapy development

## Abstract

Pseudoxanthoma elasticum (PXE), the prototype of heritable ectopic mineralization disorders, manifests with deposition of calcium hydroxyapatite crystals in the skin, eyes and arterial blood vessels. This autosomal recessive disorder, due to mutations in *ABCC6*, is usually diagnosed around the second decade of life. In the spectrum of heritable ectopic mineralization disorders are also generalized arterial calcification of infancy (GACI), with extremely severe arterial calcification diagnosed by prenatal ultrasound or perinatally, and arterial calcification due to CD73 deficiency (ACDC) manifesting with arterial and juxta-articular mineralization in the elderly; the latter disorders are caused by mutations in *ENPP1* and *NT5E*, respectively. The unifying pathomechanistic feature in these three conditions is reduced plasma levels of inorganic pyrophosphate (PPi), a powerful endogenous inhibitor of ectopic mineralization. Several on-going attempts to develop treatments for these conditions, either with the goal to normalize PPi plasma levels or by means of preventing calcium hydroxyapatite deposition independent of PPi, are in advanced preclinical levels or in early clinical trials. This overview summarizes the prospects of treatment development for ectopic mineralization disorders, with PXE, GACI and ACDC as the target diseases, from the 2020 vantage point.

## 1. Introduction

### 1.1. Clinical Features of Pseudoxanthoma Elasticum

**Figure 1:**
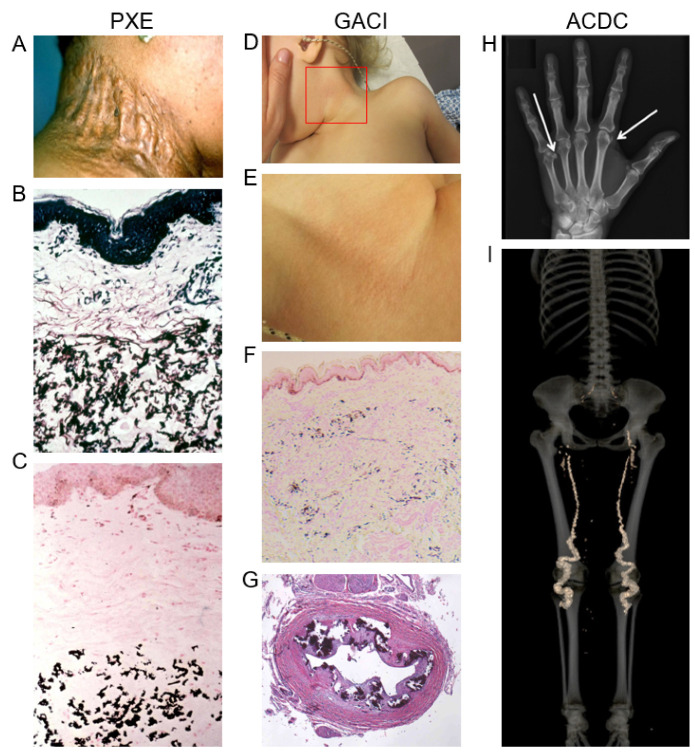
**Clinical features and ectopic mineralization in pseudoxanthoma elasticum (PXE), generalized arterial calcification of infancy (GACI), and arterial calcification due to CD73 deficiency (ACDC).** PXE is characterized by late-onset cutaneous manifestations, including loose and inelastic skin (**A**). Biopsy of the affected area of skin reveals accumulation of pleomorphic elastotic material in mid dermis as visualized by Verhoeff-van Gieson stain (**B**). The aberrant elastotic material demonstrates calcification, as shown by von Kossa stain (**C**). Some patients with GACI manifest with early cutaneous findings similar to PXE (**D**; boxed area in D is enlarged in **E**), and histopathology reveals evidence of ectopic mineralization of skin elastic fibers in the dermis (**F**). In classic cases of GACI, the patients demonstrate severe vascular mineralization with intimal hypoplasia (**G**), and the patients often die during the early postnatal period. ACDC affects primarily elderly individuals with periarticular calcification (**H**, arrows), and extensive vascular calcification in the lower extremities (**I**). (The figures were adopted, with permission, from the following publications: [2,3,4].

Pseudoxanthoma elasticum (PXE) is the prototype of heritable multisystem ectopic mineralization disorders with protean manifestations. PXE is in most cases initially diagnosed with characteristic skin findings, consisting of yellowish papules on the flexural predilection sites, including the sides of the neck, and axillary and inguinal areas. These lesions tend to coalesce into large plaques of inelastic, leathery skin with sagging and loss of recoil [1,2] (Figure 1; Table 1). Histopathology of the skin demonstrates accumulation of the pleomorphic elastotic material in the mid dermis as demonstrated by elastin specific stains (Verhoeff-van Gieson or Orcein). This aberrant elastotic material becomes calcified as demonstrated by transmission electron microscopy or by histopathologic staining with calcium phosphate stains (von Kossa or Alizarin red). While the skin manifestations can be extensive, expanding to cover most of the body, the cutaneous findings are primarily of cosmetic concern.

The diagnosis of PXE, which is based on cutaneous findings with characteristic histopathology, as well as ocular findings, combined with family history and/or demonstration of pathogenic sequence variants in the *ABCC6* gene, implies that the patients are susceptible for development of serious ocular and cardiovascular manifestations with high degree of morbidity and occasional mortality [5,6]. The characteristic ocular findings consist of angioid streaks reflecting breaks in the calcified Bruch’s membrane, an elastic sheath behind the pigmented retina, which allow capillary neovascularization to the retina, often associated with bleeding and scarring. The changes in the retina can lead to progressive loss of visual acuity and development of legal blindness in individuals in their 40s unless treated appropriately with vascular endothelial growth factor (VEGF) antagonists. The PXE patients also develop vascular complications due to mineralization of the arterial blood vessels, particularly in the lower extremities, intermittent claudication being the cardinal symptom. Clinical manifestations of vascular involvement, although less frequent, also include hypertension and occasional bleeding of the gastrointestinal vessels. In some families, there can be severe vascular involvement, including development of arterial aneurysms and occasional early myocardial infarcts and strokes.

### 1.2. Genetics and Pathophysiology of PXE

PXE is an autosomal recessive disorder with complete penetrance. Earlier suggestions of autosomal dominant inheritance in some families have been discounted by more recent genetic analyses, and occasional demonstrations of inheritance in two subsequent generations have been shown to reflect pseudodominance in consanguineous families [7]. The majority of PXE patients harbor biallelic mutations in the *ABCC6* gene, which encodes an efflux transporter protein ABC-binding cassette subfamily C, member 6 (ABCC6), expressed primarily in the liver and to a lesser extent in the proximal tubules in the kidneys [8,9]. ABCC6 is not expressed in tissues demonstrating ectopic mineralization or in the principal cells of such tissues, as for example dermal fibroblasts. Under physiological conditions, ABCC6 facilitates transport of adenosine triphosphate (ATP) from the hepatocytes to the extracellular milieu where ATP is readily converted to adenosine monophosphate (AMP) and inorganic pyrophosphate (PPi) [10,11]. The latter is a powerful physiological antimineralization factor which interferes with hydroxyapatite crystal growth by binding to the initial nucleus of mineralization and preventing calcium phosphate deposition. In patients with PXE with nonfunctional ABCC6, the plasma levels of PPi are reduced to about 40% of the level in normal healthy individuals, which is reproducibly in the range of 1 µM measured by using a number of analytical techniques [12,13]. Reduced PPi levels then allow the growth of calcium hydroxyapatite crystal deposits on the surfaces of the extracellular matrix, particularly the elastic structures. It should be noted that in PXE patients, ectopic mineralization takes place in essentially all tissues with elastic structures, yet clinical manifestations are limited to the skin, eyes, and the arterial blood vessels [14].

The PPi concentration in plasma is regulated by a number of factors. First, as indicated, the majority of PPi is derived from enzymatic hydrolysis of ATP by ENPP1, an ectonucleotide pyrophosphatase/phosphodiesterase generating AMP and PPi. However, since in complete absence of functional ABCC6, as in most cases with PXE, there is a residual of 40% of PPi in plasma, apparently reflecting other sources of ATP which can serve as a substrate for ENPP1 in extrahepatic tissues. At the same time, circulating PPi has a relatively short half-life, estimated to be approximately 42 min [15,16], due to its enzymatic hydrolysis to inorganic phosphate (Pi) by tissue nonspecific alkaline phosphatase (TNAP). The activity of this enzyme is physiologically inhibited by adenosine which is derived from further hydrolysis of AMP by another ectonucleotidase CD73 encoded by the *NT5E* gene [17]. Since in the absence of functional ABCC6, AMP levels as a substrate are also reduced, there is a reduction of adenosine plasma levels leading to reduced inhibition of TNAP activity, thus collectively leading to reduced PPi plasma levels in PXE by different mechanisms.

### 1.3. Related Ectopic Mineralization Disorders

While PXE is characteristically of late onset and a slowly progressive disorder, a few cases with characteristic features of PXE have been described in early infancy, diagnosed as pediatric PXE. However, another disease, generalized arterial calcification of infancy (GACI) is a severe ectopic mineralization disorder with primarily vascular calcification, manifesting very early on, often diagnosed by routine prenatal ultrasound at the 14th-15th weeks gestation (Figure 1; Table 1). These patients are born with extensive vascular mineralization, neointimal hyperplasia and cardiovascular insufficiency, and without treatment, these children frequently die within the first year of life from cardiovascular complications [18]. Characteristic cases of GACI, designated as GACI-1, with early onset ectopic mineralization, harbor biallelic loss-of-function mutations in the *ENPP1* gene, and their circulating PPi levels, as deduced from the corresponding *Enpp1^−/−^* mouse models [19], are essentially zero, allowing early aberrant mineralization to take place with serious phenotypic consequences [20,21]. Interestingly, some of the GACI patients, designated as GACI-2, have been shown to harbor mutations in the *ABCC6* gene, instead of *ENPP1*, demonstrating a genotypic and early phenotypic overlap between GACI and PXE [22,23]. 

**Figure 2:**
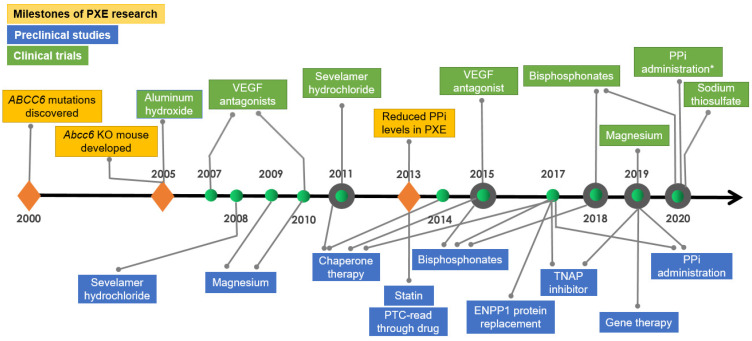
**Milestones in PXE research, preclinical studies, and early clinical trials**. The events are reported in chronological order and refer to the year of publication, except inorganic pyrophosphate trial (asterisk), which is registered as a clinical trial at early stages of development.

A third example of ectopic mineralization is arterial calcification due to CD73 deficiency (ACDC), a late-onset Mendelian disorder affecting arterial blood vessels by ectopic mineralization in the lower extremities and in periarticular tissues [17] (Figure 1; Table 1). ACDC is a very rare condition, and only a few families have been carefully examined for genetics and underlying mutations. The genetic basis of this condition resides in the *NT5E* gene, and in the absence of the CD73 activity, the adenosine levels are reduced resulting in activation of TNAP and again contributing to the reduced PPi plasma levels [3,24]. Thus, the unifying pathomechanistic feature in these three heritable ectopic mineralization disorders, viz., PXE, GACI, and ACDC, revolves around reduced PPi concentrations in the plasma, and the overall levels of this molecule, in general terms, translate to the severity and the average age of onset of the disease. It should be noted that a number of contributing factors, besides the primary genetic defects, can modulate the phenotype in different families with distinct mutations and even between affected members within the same family, clinically recognized as considerable intra- and interfamilial phenotypic heterogeneity. Such contributing factors have been postulated to include modifier genes, epigenetic factors, and environmental and lifestyle variables, such as exercise and diet [25]. Nevertheless, there is no effective treatment currently available for systemic mineralization in these three conditions, but several recent preclinical developments and early clinical trials hold promise for potential treatments in the near future (Table 2). Among these efforts, PXE is the most studied disease for therapeutics development (Figure 2).

### 1.4. Development of Animal Models

Much of the recent progress in development of therapeutic modalities for ectopic mineralization disorders, primarily focusing on PXE, has been based on preclinical testing in animal models. Following the demonstration of mutations in the *ABCC6* gene in patients with PXE, animal models consisting of *Abcc6* gene knockout (*Abcc6^−/−^*) in mice were developed [57,58]. These animals recapitulate the features of human PXE by relatively late onset of mineralization in the skin, eyes, and the arterial blood vessels. Subsequently, a number of inbred mouse strains with a spontaneous mutation in the *Abcc6* gene were identified, allowing investigation of phenotypic variability in mice with the same *Abcc6* allelic mutation on different genetic strain backgrounds [59,60]. In addition to mouse models, a rat model for PXE has also been developed by inactivation of *Abcc6* by zinc finger nuclease technology; this larger rodent model allows more detailed pathophysiological studies, such as liver and kidney perfusions, to be conducted [61]. These rodent models have served as a platform for a number of preclinical studies, some of which have led to early clinical trials (Table 2). 

A number of animal models are also available to study GACI caused by *ENPP1* mutations. The genetically engineered *Enpp1^−/−^* mice display vascular calcification phenotypes similar to human GACI [62]. The *Enpp1^asj^* and *Enpp1^asj−2J^* mice, both developed at The Jackson Laboratory, Bar Harbor, ME, display phenotypic features of stiffening of the joints, hence “ages with stiffened joints” (*asj*). These mice harbor a missense mutation, p.V246D, and a large deletion/insertion, respectively, in the *Enpp1* gene [63,64]. The “tip toe walking” (*Enpp1^ttw^*) mouse, a spontaneous mutant harboring a stop codon mutation p.G568* in the *Enpp1* gene, exhibits ectopic mineralization phenotype similar to those in *Enpp1^−/−^* mice [65]. 

A mouse model developed in 2004 to study tubuloglomerular feedback-dependent responses, was recently recognized to have features of ACDC. This mouse, developed by ablation of the *Nt5e* gene, showed profound mineralization of the costochondral junctions, juxta-articular joint capsules as well as ligaments adjacent to the bony structures, features in patients with ACDC [24,66]. These mice, however, do not develop vascular mineralization as characteristically seen in human disease. 

## 2. Therapy Development for Ectopic Mineralization Disorders

### 2.1. Dietary Magnesium

Early literature suggested that excessive dietary intake of dairy products in the early childhood and adolescence may result in more severe presentation of PXE later in life, and this was taken as an indication of the role of dietary calcium in ectopic mineralization, with a corresponding advice for the PXE patients to limit their calcium intake [1]. To provide scientific basis for this recommendation, one of the early studies on PXE mouse models tested the effects of a number of dietary minerals on the severity of the disease. These studies demonstrated that dietary calcium did not have any effect on ectopic mineralization in the mouse model of PXE. However, it was noted that increased magnesium content of the diet fed to the mice had a profound effect on the degree of mineralization. Specifically, increase of the magnesium content by 5-fold over the regular diet completely abolished the ectopic mineralization in the PXE mice [27,28,29]. Elevated dietary magnesium by 5-fold during pregnancy and postnatal life also prevented the ectopic mineralization in the *Enpp1^asj^* mice [30]. Conversely, reduction of the magnesium content to 20% of the control diet resulted in significant acceleration of the mineralization process [67]. The mechanism by which elevated dietary magnesium prevents ectopic mineralization involves competition of magnesium for calcium in complexing with inorganic phosphate. Since magnesium phosphate complexes are more soluble than calcium phosphate particles, less hydroxyapatite precipitation and crystal formation occurs.

Based on these preclinical observations, a two-year clinical trial supplementing PXE patients’ diet with magnesium was initiated. The recommendation of the United States Food and Drug Administration for daily magnesium content of diet is 420 mg for adult male and 320 mg for adult female. The first year of this placebo-controlled trial consisted of placebo or supplementation of diet with magnesium oxide (MgO), 800 mg twice a day (a total of 1000 mg of elemental magnesium), with 22 individuals enrolled in each group [50]. The effect of the treatment was monitored by examination of the extent of skin lesions and obtaining skin biopsies for assessment of the degree of calcification at the beginning and the end of one year treatment period, the primary endpoint being calcification of skin elastic fibers. At the end of a double-blinded one year period, all 44 individuals were encouraged to continue on supplementary magnesium intake, 2500 mg of MgO (1500 mg of elemental magnesium). 

During the double-blinded, placebo-controlled phase, the magnesium group had a decrease, yet not reaching statistical significance, in calcification of the skin elastic fibers, whereas the placebo group did not demonstrate any changes. Setting a 30% decrease in elastic fiber calcification as a secondary endpoint, 36% of patients in the treatment group were responders compared with only 14% of patients in the placebo group. The authors concluded that, although not statistically significant, this trend might be clinically significant [50]. A similar trend was noted in assessment of the cutaneous lesions, but no significant treatment effect was observed in the ophthalmological outcomes. The investigators pointed out that this study had limitations that likely contributed to the lack of statistical significance, including the relatively small cohort size. In addition, the dose of magnesium was limited because of concern of gastrointestinal side effects, particularly diarrhea. Despite these limitations, the results highlight a promising trend showing reduction in calcification of skin elastic fibers while on magnesium supplementation. A larger study cohort could further elicit the value of magnesium supplementation as an antimineralization therapeutic either as a monotherapy or in combination with other approaches. 

### 2.2. Phosphate Binders

One of the observations in the initial preclinical studies using *Abcc6* knockout mice as a platform to study the modulation of the PXE phenotype was that dietary phosphate had a profound effect on the degree of mineralization. Specifically, increasing the phosphate content of the diet by 2-fold clearly accelerated the mineralization in this mouse model, particularly when coupled with magnesium content reduced to 20% of the control diet [67]. This so-called “acceleration diet” (2-fold increase in Pi, 20% Mg^2+^, and 22-fold increase in vitamin D content) has been used to accelerate the mineralization in rodent models of PXE, thus speeding up the preclinical trials towards development of treatment modalities. 

Considering that phosphate promotes the mineralization process, limited early clinical trials have been performed with phosphate binders as pharmacological agents limiting the intestinal absorption of phosphate in patients with PXE. In the first early study, six patients were treated with aluminum hydroxide and evaluated by skin photography and lesion assessment [47]. Repeated skin biopsies were also performed on clinically improved target sites, and ophthalmologic evaluation was performed at yearly intervals. Of the six patients, three showed apparent clinical improvement of skin lesions with histopathologic regression of the disease. No deterioration of eye involvement was noted. 

This relatively small study was expanded to include 40 patients with PXE randomized to receive either sevelamer hydrochloride (800 mg by mouth 3 times daily) or placebo up to one year [49]. During the second year, all patients received sevelamer hydrochloride. A decrease in the calcium score was noted during the first year both in the treatment and placebo group, and in the second year the calcium scores decreased further. The mean clinical scores also improved in the sevelamer treated patients more significantly than in the placebo group. The authors concluded that sevelamer hydrochloride can produce a reduction in both calcification levels and clinical scores, but this difference was not statistically significant compared with placebo. The improvement in placebo group could possibly reflect the fact that both the placebo and the active drug preparations contained magnesium stearate as an inactive ingredient, which may have played a confounding role in this study. 

### 2.3. Atorvastatin

ABCC6 has been suggested as a new player in cellular cholesterol and lipoprotein metabolism, as described for other ABC transporters [68]. Early studies suggested that cholesterol-lowering drugs, such as atorvastatin, was able to ameliorate the extent of ectopic mineralization in the *Abcc6^−/−^* mouse model [31]. In contrast, an atherogenic diet induced hypercholesterolemia and steatosis accompanied by increased mineralization in the soft connective tissues in the same mouse model [69]. These findings have clinical relevance for the management of PXE in humans.

### 2.4. Inorganic Pyrophosphate

Following the demonstration of the critical role of reduced PPi plasma levels in the pathomechanism of PXE, a logical thought was to augment the PPi levels by oral administration. This notion was initially discarded because the earlier literature had suggested that PPi does not get absorbed from the intestine [70]. In fact, PPi, which is commonly used by the food industry as an emulsifier, was considered to be completely inactive when administered by mouth. However, subsequent studies in mice and human volunteers clearly documented that a small fraction, in the order of 0.3% of the total dose of tetrasodium-PPi administered orally, is absorbed and is able to elevate the plasma PPi levels [16,70]. Subsequent studies demonstrated that oral administration of tetrasodium-PPi inhibited connective tissue mineralization in both *Abcc6^−/−^* and *Enpp1^asj^* mice [16]. Further modifications, including use of a disodium-PPi, was tested in individuals with PXE, without apparent gastrointestinal side effects, potentially providing a new treatment modality for PXE and related ectopic mineralization disorders [71]. Based on these studies, a disodium-PPi absorption trial in PXE patients is under way (NCT04441671; last updated 22 June 2020).

### 2.5. Bisphosphonates

Bisphosphonates are stable analogs of PPi in which the oxygen linkage between phosphonate (PO3) groups has been replaced by a carbon molecule, thus providing stability to the molecule against hydrolysis by TNAP [72]. In addition, bisphosphonates have two side groups (R1, R2) which can be chemically modified, providing unique pharmacokinetic characteristics, pharmacologic profiles, and potency. Bisphosphonates have been developed as drugs to counteract osteoporosis, but in principle, they have two different effects: (a) they can serve as an antimineralization compound, and (b) they promote bone calcification by being powerful antiosteoclastic molecules. The initially synthesized bisphosphonates, such as etidronate, have a higher relative antimineralization activity as compared to the antiosteoclastic activity. However, the more recently synthesized, N-containing bisphosphonates, such as alendronate, which are multiple orders of magnitude more powerful than etidronate, favor the antiosteoclastic activity. Based on these considerations, etidronate, as a stable PPi analog, was initially tested in the mouse models for PXE for its efficacy as an antimineralization factor. The results clearly demonstrated that etidronate, although at relatively high concentrations, were able to prevent ectopic mineralization in the mouse model of PXE [36]. Similar observations were also made in GACI mice with deficient ENPP1 activity. In the latter mice, which also demonstrate extensive bone abnormalities, correction of the defective bone mineralization was noted as a result of etidronate treatment [38,39]. Interestingly, administration of alendronate to these mouse models did not demonstrate significant antimineralization activity, consistent with its characteristics primarily as an antiosteoclastic compound.

Based on the preclinical studies on bisphosphonates, a clinical trial treating PXE patients with etidronate was initiated, and this double-blinded trial included 74 patients, evenly divided into the etidronate and placebo groups (Clinical Trial Code NTR5180) [51]. The patients were cyclically administered placebo or etidronate, 20 mg per kg/per day for 2 weeks every 12 weeks. The primary outcome was ectopic mineralization of the femoral arterial wall as quantified by ^18^fluoride positron emission tomography (^18^F-NaF-PET) and computed tomography (CT) scans. Although there was considerable variability between individuals, the investigators demonstrated that during the initial 12-month treatment period, arterial calcification in the lower extremities decreased 4% in the etidronate group and increased 8% in the placebo group. Etidronate treatment was also associated with significantly fewer subretinal neovascularization events, leading to the conclusion that etidronate may be helpful for treatment of patients with PXE. Thus, the results suggested that etidronate can halt the progression of femoral artery calcification, and possibly facilitate removal of some of the existing hydroxyapatite lesions [51,73].

As a follow-up of the initial report, the investigators have published a comprehensive survey of all vasculature with respect to ectopic mineralization in these patients and the effect of etidronate treatment [52]. As a prespecified post-hoc analysis of the initial study, calcification mass was quantitated in a number of arterial blood vessels, including thoracic and abdominal aorta, using CT as a baseline and after one year of treatment with etidronate or placebo. The results revealed that etidronate significantly halted progression of calcification in all vascular beds examined, except for the coronary arteries. These results, therefore, revealed that etidronate treatment can halt systemic arterial calcification in PXE, emphasizing its potential usefulness for treatment of this disorder. However, as the authors noted, further research must assess the long-term safety and efficacy of etidronate on clinical outcomes.

The efficacy of bisphosphonates for treatment of GACI has also been tested in limited number of patients, with encouraging results. Specifically, a retrospective observational analysis of 55 patients with GACI revealed survival beyond infancy with etidronate therapy [18], which was corroborated by another study reporting that 15 of 22 GACI patients who received etidronate treatment survived beyond infancy [74]. Bisphosphonates have also been reported to be accompanied by severe side effects, particularly on the development of bones [75].

In addition to PXE and GACI, an etidronate trial is currently underway to investigate whether etidronate is a safe and effective treatment for ACDC (NCT01585402; last update 10 July 2020), which is also accompanied by reduced plasma PPi levels.

### 2.6. Tissue Nonspecific Alkaline Phosphatase (TNAP) Inhibitors

One approach towards increasing plasma PPi levels involves inhibition of TNAP, the principal enzyme that facilitates degradation of PPi. The effects of TNAP inhibition, genetically and pharmacologically, have been tested in both *Abcc6^−/−^* and *Enpp1^asj^* mice. In a genetic model, the compound *Alpl^+/−^;Abcc6^−/−^* mice, with approximately 50% reduced TNAP levels, had dramatic reduction of ectopic mineralization as compared to age-matched *Alpl^+/+^;Abcc6^−/−^* mice, despite unchanged plasma PPi levels [44]. In addition, administration of SBI-425, a small molecule inhibitor of TNAP, prevented ectopic mineralization in the *Abcc6^−/−^* mice [44,45]. Several small molecules modified from SBI-425 with several folds higher potency and better pharmacological properties are currently being developed.

### 2.7. ENNP1 Enzyme Replacement Therapy

With the focus on efforts to normalize the plasma PPi levels in patients with ectopic mineralization disorders, some recent studies have concentrated on ENPP1, the key enzyme catalyzing the hydrolysis of ATP to AMP and PPi. In patients with mutations in the *ENPP1* gene, this enzymatic activity can be very low or essentially absent, and replacement therapy with recombinant enzyme is expected to result in normalization of the PPi plasma levels with stabilization of the disease. Initial studies using *Enpp1^asj^* mice, in fact, revealed that administration of recombinant ENPP1 protein counteracted the phenotype, and specifically, the treatment prevented mortality, vascular calcification, and improved blood pressure and cardiovascular function [42]. Thus, ENPP1 replacement therapy would be expected to correct or counteract the development of the GACI phenotype in humans as well.

ENPP1 replacement therapy has also been suggested for PXE. While the primary genetic defect in this disorder resides in the *ABCC6* gene, cellular studies have suggested that the activity of ENPP1, which resides downstream of ABCC6 in the pathway in generation of PPi, is also reduced, and ENPP1 supplementation could augment PPi production from ATP in extrahepatic tissues independent of ABCC6. Genetic overexpression of human ENPP1 normalized plasma PPi levels and significantly prevented ectopic mineralization in the *Abcc6^−/−^* mice, suggesting that the ENPP1 replacement therapy is also applicable to patients with PXE [41].

### 2.8. Prospects of Allele-Specific Therapies

A number of potential treatment approaches for PXE have also been examined at either utilizing PXE mouse models or cells with *ABCC6* mutations at early preclinical level, but these studies have not yet led to clinical trials. Some of these approaches are allele-specific, i.e., applicable only to a subset of patients with specific types of mutations. These approaches include read-through of premature termination codon (PTC) in the *ABCC6* gene with the prototypic nonsense codon read-though-inducing drug, PTC124. Utilizing human HEK293 cells transfected with human ABCC6 expression constructs harboring different PXE-associated nonsense mutations, these studies demonstrated that PTC124 was able to facilitate PTC read-through, but the efficacy varied not only with dose but also with the sequence context of the stop codon [35]. Thus, these results suggested that read-through of nonsense mutations in *ABCC6* by PTC124 may have potential for pharmacologic treatment of some patients with PXE, and development of this, and other allele-specific drugs, emphasize the importance of knowledge of the precise sequence information of the mutant alleles.

Another allele-specific treatment modality, limited to a subgroup of patients with distinct *ABCC6* missense mutations, deals with molecular chaperones for correcting the intracellular trafficking defect of the mutant protein. Specifically, molecular studies have identified PXE mutations resulting in synthesis of catalytically active ABCC6 protein, but its intracellular transport to the basolateral surface of hepatocytes is perturbed, leading to cytoplasmic accumulation. In case of some of these mutations, the intracellular trafficking defect can be corrected by treatment of cells with a chaperone molecule, 4-phenylbutyrate, which is already in clinical use for urea cycle disorders and thalassemia, thus possibly offering a treatment modality for selected patients with PXE [15,32,33,34].

### 2.9. Prospects of Gene Therapy

A considerable amount of investigation has been devoted for development of gene therapy for metabolic disorders with the primary defect in the liver, and some of these are already in clinical trials, as exemplified by hemophilia [76]. Preliminary attempts have been made to develop gene therapy for PXE, with the goal to introduce a functional copy of the *ABCC6* gene into the *Abcc6^−/−^* mice. For this purpose, an adenovirus construct into which a full-length *ABCC6* cDNA had been incorporated was injected into the tail vein of these mice in a manner that the vector reaches liver [46]. The results demonstrated strong hepatic expression of the transgenic human ABCC6, and this expression was sustained up to 4 weeks after a single injection. However, since this model employed adenovirus which has a high immunogenic potential, an immunologic suppression of the mouse was required, a situation complicating the development of this vector for human application. Viral vectors with less immunogenicity, such as adeno-associated virus, can be tested in human trials.

### 2.10. VEGF Antagonists

To prevent visual impairment caused by choroidal neovascularization in patients with PXE, intravitreal injections of anti-VEGF compounds are now in routine use with considerable success [77,78]. The pharmaceutical agents, bevacizumab and ranibizumab, antagonists of VEGF, which were initially tested, hold promise for long-term control of choroid neovascularization in PXE, and intravitreal injections of other VEGF antagonists, for example aflibercept, have been recently presented to be effective in prevention of loss of visual acuity [55]. It should be noted that the VEGF antagonists target only neovascularization, a consequence of calcification of the Bruch’s membrane, and they do not antagonize the mineralization process *per se*.

### 2.11. Potential Treatments for Reversal of the Existing Calcium Hydroxyapatite Crystals

One of the considerations regarding the treatment approaches currently under development, as summarized above (Table 2), relates to the fact that they are primarily preventing further mineralization by arresting the hydroxyapatite crystal growth, and it is unclear whether they are able to reverse the existing mineralization as a result of endogenous turnover of the tissues with mineral deposits. This is an obvious concern to the individuals affected by these conditions, because already at the time of diagnosis, they have significant calcium deposits which explain their clinical features leading to correct diagnosis. The future efforts should, therefore, also focus on pharmacological approaches which would dissolve the existing mineral deposits. Such possibilities include systemic administration of pharmacologic agents which would elicit dissolution of calcium phosphate complexes, such as calcium chelator citrate, now in clinical use for prevention of calcium-containing kidney stones [79]. In this context, kidney stones, a major public health challenge, have also been reported as a frequent manifestation in patients with PXE. In one study, a 39.8% prevalence of kidney stones in 113 French PXE patients was reported [80]. Furthermore, in a recent study on a cohort of 563 PXE patients residing in the United States, 23% of them reported to have a history of kidney stones, a significant increase compared to 9.2% in the US general population [81]. A number of novel therapeutic agents to counteract ectopic mineralization are currently in preclinical or early clinical testing. Such molecules include inositol hexaphosphate (INS-3001) and myo-inositol hexaphosphate (SNF472), potential inhibitors of ectopic mineralization [82,83] but whether they will elicit reversal of the existing mineral deposits is currently unknown. In addition, although transient, the efficacy of intravenous sodium thiosulfate in dissolution of ectopic calcification was demonstrated in one PXE patient with severe early-onset manifestations [56]. Finally, in addition to systemic therapies, development of targeting strategies to limit the delivery of the dissolving agents to arterial elastic fiber associated mineral deposits has been reported using conjugates of antielastin antibody nanoparticles coupled with EDTA, a powerful calcium solubilizing chemical [84]. Further work is necessary to investigate the preclinical and clinical potential of these approaches for removal of existing mineralization in PXE, GACI, and ACDC.

## 3. Conclusions and Future Perspectives

Our understanding of the genetic basis and molecular details of the pathomechanisms of heritable ectopic mineralization disorders has tremendously expanded over the past decade, and this knowledge has now identified a number of potential targets for treatment development [2]. Specifically, the common pathomechanistic feature with heritable ectopic mineralization disorders discussed in this review, PXE, GACI, and ACDC, revolves around the role of reduced PPi plasma levels, with the notion that normalization of PPi could prevent further ectopic mineralization, potentially stabilizing the disease. Such normalization could potentially include oral administration of PPi or its derivatives with different pharmacokinetics which might lead to sustained elevation of PPi. TNAP inhibitors, which would prevent degradation of PPi, could also lead to elevated PPi plasma levels. One of the stable PPi analogs, etidronate, is already in clinical trials and has been shown to prevent the ectopic mineralization of arterial blood vessels in PXE [51,52]. In addition, etidronate has been tested in a few selected cases of GACI to examine whether administration of etidronate to pregnant mothers and to the affected infants postpartum would be reducing the cardiovascular complications [18,53]. If successful, some of these treatments focusing on PPi levels could be complemented with treatment modalities counteracting other mechanisms of mineralization, including magnesium or phosphate binder administration, which in combination could prevent the progression of the disease. If arrest of further progression of the mineralization is achieved by these treatment modalities, the implication is that the treatment should be started as early as possible at the time the diagnosis has been established. In this context, in case of family history of PXE, potentially susceptible individuals, particularly unaffected siblings of an affected individual, could be tested by mutation analysis for presymptomatic diagnosis to include or exclude the possibility of the disease at a later state, a situation that would allow preventive treatment before the symptoms develop [85].

## Figures and Tables

**Table 1:** Clinical and genetic features of PXE, GACI, and ACDC.

Characteristics	PXE	GACI	ACDC
*Genetic basis*			
Mutated genes	*ABCC6*, *ENPP1*	*ENPP1*, *ABCC6*	*NT5E*
*Clinical features*			
Yellowish skin papules	Yes	Rare	No
Mineralization in skin lesions	Yes	Yes	No
Retinal angioid streaks	Yes	Rare	No
Vascular calcification	Yes	Yes	Yes (lower extremity)
Periarticular calcification	No	Yes	Yes
Hearing loss	No	Yes	No
Hypophosphatemic rickets	No	Yes (survivors)	No
Age at onset	Adolescence, early adulthood	Pre- and perinatal onset	Adult onset
Life expectancy	Mostly normal	Demise common <6 months	Normal
*Plasma PPi levels*	Reduced to ~40%	Reduced to 0	Reduced to ~50%

PXE, pseudoxanthoma elasticum; GACI, generalized arterial calcification of infancy; ACDC, arterial calcification due to deficiency of CD73.

**Table 2:** Interventional preclinical studies and early clinical trials for PXE, GACI, and ACDC, published and on-going.

Treatment	Rationale/Target	Diagnosis	Publications
*Preclinical trials*			
Sevelamer hydrochloride	Phosphate binder	PXE	[26]
Magnesium	Competes with calcium to prevent calcium hydroxyapatite crystal formation	PXE	[27,28,29]
		GACI	[30]
Statin	Lipid-lowering drug	PXE	[31]
Chaperone therapy	Corrects the trafficking defect of misfolded ABCC6 protein	PXE	[15,32,33,34]
PTC-read through drug	Read-through of PTC codons	PXE	[35]
Bisphosphonates	Stable nonhydrolyzable PPi analogs	PXE	[15,36,37]
		GACI	[38,39]
PPi administration	Correction of reduced plasma PPi levels in PXE and GACI	PXE	[15,16,40]
		GACI	[16]
ENPP1 protein replacement	ENPP1 is the principal enzyme that generates PPi	PXE	[41]
		GACI	[42,43]
TNAP inhibitor	TNAP is the enzyme that breaks down PPi	PXE	[44,45]
		GACI	[44]
Gene therapy	*ABCC6* gene therapy to reconstitute hepatic ABCC6	PXE	[46]
*Clinical trials*			
Aluminum hydroxide ^1)^	Phosphate binder	PXE	[47]
VEGF antagonists ^2)^	Bevacizumab and ranibizumab, inhibitors of vascular endothelial growth factor for the treatment of choroidal neovascularization	PXE	[48]
Sevelamer hydrochloride ^3)^	Phosphate binder	PXE	[49]
Magnesium ^4)^	Prevention of ectopic mineralization	PXE	[50]
Bisphosphonates ^5)^	Stable nonhydrolyzable PPi analogs	PXE	[51,52]
		GACI	[18,53,54]
		ACDC	NCT01585402 *
VEGF antagonist ^6)^	Aflibercept, an inhibitor of vascular endothelial growth factor for the treatment of choroidal neovascularization	PXE	[55]
Sodium thiosulfate ^7)^	Antioxidant properties and chelation of calcium to form soluble calcium thiosulfate complexes	PXE	[56]
PPi administration	Correction of reduced plasma PPi levels in PXE and GACI	PXE	NCT04441671 *

PXE, pseudoxanthoma elasticum; GACI, generalized arterial calcification of infancy; ACDC, arterial calcification due to deficiency of CD73. * ClinicalTrials.gov Identifier; ^1)^ Six PXE patients, 1-year follow-up. Three patients showed significant clinical improvement and histopathologic regression of calcification in the skin lesions. No deterioration of eye disease was observed in any of the six patients; ^2)^ Long-term results of intravitreal antivascular endothelial growth factors, bevacizumab and ranibizumab, in the management of choroidal neovascularization in nine PXE patients with angioid streaks. During the mean follow-up period of 28.6 months, visual acuity was either improved or stabilized in all patients without complications; ^3)^ Randomized, double-blind, placebo-controlled, prospective clinical study in 40 patients with PXE. While reduction in both calcification levels and clinical scores of the skin were noted, the differences were not statistically significant compared with placebo; ^4)^ The NCT01525875 magnesium trial was a randomized, double-blind, placebo-controlled prospective trial that evaluated the effect of oral magnesium oxide (MgO) versus placebo on the skin and eyes in 44 PXE patients. Although not statistically significant, the treatment group was found to have decreased elastic fiber calcification in the skin. No significant treatment effect was observed in the ophthalmologic outcomes; ^5)^ The Treatment of Ectopic Mineralization in Pseudoxanthoma Elasticum trial (NTR5180) tested the effects of etidronate in 74 PXE patients with leg arterial calcifications (mean age: 57 years, range 48–66) randomly assigned to etidronate or placebo. During 12 months of follow-up, etidronate reduced arterial calcification and subretinal neovascularization events without important safety issues; ^6)^ The NCT02537054 trial was a 12-month prospective, open-label, uncontrolled, nonrandomized interventional clinical trial testing the effects of 2 mg intravitreal aflibercept for treatment of choroidal neovascularization (CNV) secondary to angioid streaks, in 15 PXE patients (mean age: 53 years, range 22–65). After 12 months of treatment, aflibercept improved or stabilized best corrected visual acuity (BCVA), central retinal thickness (CRT), retinal sensitivity, and vision-related quality of life in the majority of patients; ^7)^ Intravenous sodium thiosulfate was administered in a young patient with PXE-like syndrome. This 11-year old patient, with severe early-onset systemic calcifications occurring in the skin and in the arteries, demonstrated radical improvement in asthaenia, anorexia and pre-/postprandial pain and calcific stenosis after six months of treatment.

## Data Availability

Not applicable.
